# Coronavirus (COVID-19) infection rates and vaccination uptake in school pupils in England.

**DOI:** 10.23889/ijpds.v7i3.1914

**Published:** 2022-08-25

**Authors:** Joe Kelly, Alison Judd

**Affiliations:** 1 Office for National Statistics

## Objectives

COVID-19 vaccination uptake was lower in school-age children compared to older age groups. Our study aims to understand factors associated with uptake in school-age children and how vaccination programme affected infection rates in school settings.

## Approach

This study utilises existing administrative data sources to create a children’s health data asset. The core study population includes over 8 million pupils registered in English state schools whose records have been linked to NHS Test and Trace data and National Immunisation Management Service (NIMS) data. Census 2021 data will also be included enriching the data on background characteristics of each pupil. Taking into account the school attended will allow for analysis into the clustering of infections and the impact of overall school vaccination rates on infections.

## Results

Initial analyses have provided detailed insights into vaccine uptake and its impact on infection. For example, vaccination uptake was found to vary by ethnic group, with Chinese (75%) and Indian (65.7%) 12–15-year-olds most likely to be vaccinated by January 2022. Those in receipt of free school meals (FSM) were less likely to have been vaccinated than non-FSM pupils (35.9% compared with 58.9%); However, there was a wide range in vaccination rates between different schools with similar levels of deprivation.

We also found that the rate of reporting a positive test in the first half of the Autumn 2021 term was estimated to be 38% higher for unvaccinated pupils in comparison to pupils vaccinated with one dose (following 14 days after vaccination).

## Conclusion

The size and longitudinal nature of the dataset provides the potential to answer a wide variety of research questions. We must, however, be mindful of inherent biases that might occur when relying on using Test and Trace data to identify COVID-19 infections. Work is currently ongoing to expand this asset to include other data sources.

